# Changes in the gene expression profile of *Arabidopsis thaliana *after infection with *Tobacco etch virus*

**DOI:** 10.1186/1743-422X-5-92

**Published:** 2008-08-07

**Authors:** Patricia Agudelo-Romero, Pablo Carbonell, Francisca de la Iglesia, Javier Carrera, Guillermo Rodrigo, Alfonso Jaramillo, Miguel A Pérez-Amador, Santiago F Elena

**Affiliations:** 1 Instituto de Biología Molecular y Celular de Plantas, Consejo Superior de Investigaciones Científicas-UPV, 46022, València, Spain; 2 Laboratoire de Biochimie, École Polytechnique, 91128, Palaiseau, France

## Abstract

**Background:**

*Tobacco etch potyvirus *(TEV) has been extensively used as model system for the study of positive-sense RNA virus infecting plants. TEV ability to infect *Arabidopsis thaliana *varies among ecotypes. In this study, changes in gene expression of *A. thaliana *ecotype L*er *infected with TEV have been explored using long-oligonucleotide arrays. *A. thaliana *L*er *is a susceptible host that allows systemic movement, although the viral load is low and syndrome induced ranges from asymptomatic to mild. Gene expression profiles were monitored in whole plants 21 days post-inoculation (dpi). Microarrays contained 26,173 protein-coding genes and 87 miRNAs.

**Results:**

Expression analysis identified 1727 genes that displayed significant and consistent changes in expression levels either up or down, in infected plants. Identified TEV-responsive genes encode a diverse array of functional categories that include responses to biotic (such as the systemic acquired resistance pathway and hypersensitive responses) and abiotic stresses (droughtness, salinity, temperature, and wounding). The expression of many different transcription factors was also significantly affected, including members of the R2R3-MYB family and ABA-inducible TFs. In concordance with several other plant and animal viruses, the expression of heat-shock proteins (HSP) was also increased. Finally, we have associated functional GO categories with KEGG biochemical pathways, and found that many of the altered biological functions are controlled by changes in basal metabolism.

**Conclusion:**

TEV infection significantly impacts a wide array of cellular processes, in particular, stress-response pathways, including the systemic acquired resistance and hypersensitive responses. However, many of the observed alterations may represent a global response to viral infection rather than being specific of TEV.

## Background

Virus infection typically alters host's physiology, diverting almost any sort of metabolite for the production of virus-specific components, and actively manipulates antiviral defenses. As a response to viral infection, cells may compensate by over- or under-expressing certain metabolic pathways, including specific antiviral responses (e.g., the interferon or RNA silencing pathways). Taken together, all these alterations determine the strength and type of symptoms displayed by infected organisms. In the case of plant viruses, in the absence of a hypersensitive response (i.e., apoptotic cell death), cells that have successfully supported viral replication do not die but retain large amounts of viral particles while the infections spreads out through the plasmodesmata to neighboring cells until reaching the vascular system and colonizing distant susceptible tissues. The outcome of this systemic infection is the appearance of symptoms. The strength and properties of symptoms can vary widely. Even for a given pair of plant and virus species, symptoms will depend upon specific combinations of plant and virus genotypes and, of course, on environmental conditions.

Much effort has gone into identifying individual cellular traits that may change their pattern of gene expression as a direct or indirect consequence of viral infection [1]. Identifying just one of such genes was a time-consuming task. However, with the advent of DNA microarray technologies, it has now become feasible to comprehensively examine gene expression networks during plant defense response triggered by infection with viral pathogens [2-4]. Just to mention a few examples, the alterations in *Arabidopsis thaliana *gene expression profile has been analyzed in plants infected with *Tobacco mosaic virus *(TMV) [5], *Cucumber mosaic virus *[6,7], and *Turnip mosaic virus *[8]. Genes showing significant alterations in expression profiles include transcription factors, heat-shock proteins (HSP), defense-regulated genes, phytohormone biosynthesis and signaling, kinases and phosphatases, antioxidants, many different metabolic enzymes, proteases and other genes involved in protein turnover, and genes relevant for chloroplast functions among many others (reviewed in [4]).

Here we explore the altered expression profile in systemically infected leaves of *A. thaliana *ecotype L*er *infected with *Tobacco etch virus *(TEV). TEV is the type member of the *Potyvirus *genus of the *Potyviridae *family and its genome is composed by a 9.5 kb positive polarity single-stranded RNA that encodes a large ORF whose translation generates a polyprotein that is subsequently self-processed by virus-encoded proteases into 10 mature peptides [9,10]. TEV has a moderately wide host range infecting 149 species from 19 families [11], although most of its natural hosts belong to the family *Solanaceae*. In these plants TEV induces stunting and mottling, necrotic etching and malformation in leafs [11]. *A. thaliana *ecotypes vary in their susceptibility to TEV. Some ecotypes (e.g., C24 and L*er*) are fully susceptible [12,13] whereas many other (e.g., Col-0 and Ws-2) do not allow for systemic movement but support replication and cell-to-cell spread in inoculated leafs [12,13]. The particular ecotype used in this study, L*er*, shows mild symptoms associated with a low viral titer. Microarray results identified sets of genes whose expression patterns show significant alterations in TEV inoculated plants. The classification of these genes into functional categories and their putative role in disease progress will be discussed. Finally, the overlapping between these functional categories and metabolic pathways is also explored and we found that hub pathways from central metabolism are involved in several functional responses.

## Results

### Differences in transcriptional profiles

**Figure 1:**
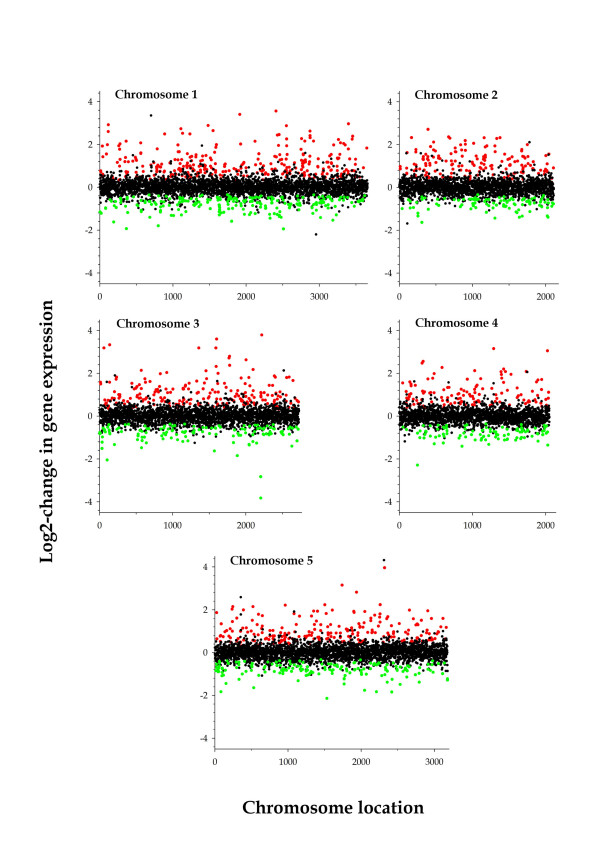
**Distribution of functional responses to TEV infection along the five chromosomes of *A. thaliana***. Reed and green dots correspond, respectively, to genes that were significantly over- or under-expressed in infected plants relative to mock-inoculated plants; black dots correspond to genes whose expression level was not affected by TEV infection. Each dot represents the median value of five independent microarray experiments.

As a preliminary analysis, we were interested in knowing whether the overall pattern of gene expression was significantly affected by TEV infection. To do so, an ANOVA model in which gene (the 13,722 valid genes in the dataset) and treatment (mock-inoculated versus infected) were treated as orthogonal fixed factors was fitted to the expression data. No overall difference existed among genes (*P *= 0.139), although infection significantly affected the levels of gene expression (*P *< 0.001). More interestingly for the purpose of this article, a significant gene-by-treatment interaction was detected (*P *= 0.012), suggesting that genes, on average, expressed differentially among non-infected and infected plants. Next, we used the SAM package [14], with a 5% false discovery rate (FDR) to identify individual genes whose expression was altered after the infection with TEV. A total of 1727 genes showed a significant alteration in their level of expression in infected plants. Indeed, significantly more genes were over- than under-expressed (1027 *vs *700; Binomial test, *P *< 0.001). Figure 1 shows the distribution of the fold-change in gene expression along the five *A. thaliana *chromosomes. Overall, no differences existed among chromosomes in the distribution of up-regulated, non-affected and down-regulated genes upon infection with TEV (homogeneity test, χ^2 ^= 14.44, 8 d.f., *P *= 0.071), suggesting that genes involved in response to TEV infection were not clustered but randomly distribute among the five chromosomes.

### Functional categorization of genes over-expressed in TEV infected plants

**Figure 2:**
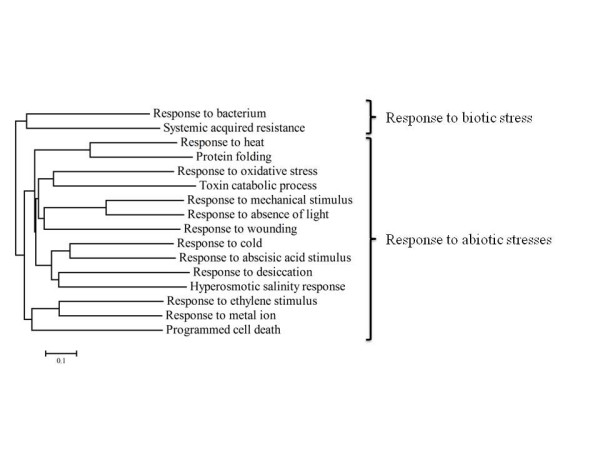
Neighbor-joining dendrogram illustrating the relationship among over-represented non-redundant GO categories obtained for genes that were up-regulated by TEV infection.

Next we sought to explore which functional categories were affected by TEV infection. To this end gene ontology (GO) enrichment analyses were performed using the FatiGO tool [15]. Table 1 shows the non-redundant functional categories significantly over- and under-represented among those genes that were significantly up-regulated upon TEV infection. A total of 16 non-redundant categories were over-represented, among these, genes related to cold response were the most abundant (25) whereas genes related to the absence of light were the less common (3). Nonetheless, different GO categories were not mutually exclusive and the same gene can be found in many different categories, according to its GO annotation. Given this non-independence among non-redundant GO categories, we can use the number of shared genes among every pair of GO categories to compute a similarity matrix [the (*i*, *j*) element of the matrix was computed according to *S*_*ij *_= 2*n*_*ij*_/(*n*_*i *_+ *n*_*j*_), where *n*_*i *_and *n*_*j *_were the genes belonging to categories *i *and *j *and *n*_*ij *_the number of genes shared among both categories] that will allow constructing a neighbor-joining tree. This tree classifies GO categories according to their interdependence. Figure 2 shows the dendrogram obtained for the over-represented categories in Table 1. Biotic and abiotic responses were clearly separated into two clusters.

**Table 1:** Gene ontology analyses of up-regulated genes

**Non-redundant GO categories**	**Level**	**Differentially** ** expressed (%)**	**Total genes in** ** the class (%)**	***P***
**Over-represented**				
Response to mechanical stimulus	4	1.07 (5)	0.03	6.36×10-^4^
Response to wounding	4	2.99 (14)	0.78	1.17×10-^7^
Response to abscisic acid stimulus	5	5.59 (24)	1.72	1.76×10-^4^
Response to bacterium	5	3.73 (16)	0.77	1.31×10-^4^
Response to cold	5	5.83 (25)	1.34	1.34×10-^6^
Response to ethylene stimulus	5	4.43 (19)	1.17	2.84×10-^4^
Response to heat	5	4.90 (21)	0.78	1.17×10-^7^
Response to metal ion	5	2.56 (11)	0.63	1.13×10-^2^
Response to oxidative stress	5	4.20 (18)	1.60	1.77×10-^2^
Hyperosmotic salinity response	6	1.81 (6)	0.27	2.79×10-^2^
Protein folding	6	6.04 (20)	2.36	1.16×10-^2^
Response to desiccation	6	1.51 (5)	0.1	5.98×10-^3^
Toxin catabolic process	6	2.42 (8)	0.24	1.14×10-^2^
Programmed cell death	7	3.61 (9)	0.76	1.33×10-^2^
Response to absence of light	7	1.20 (3)	0.03	2.75×10-^2^
Systemic Acquired Resistance	8	4.67 (7)	0.42	1.04×10-^3^
**Under-represented**				
Organelle organization and biogenesis	4	1.07 (5)	5.19	3.42×10-^4^
DNA metabolic process	5	0.70 (3)	3.56	1.62×10-^2^

Non-redundant GO categories identified as enriched among up-regulated genes in infected plants versus mock-inoculated plants. The percentages of genes belonging to each category are reported for the differentially expressed genes and for the *A. thaliana *genes present in the microarray. The absolute number of genes is reported in parenthesis for the differentially expressed set. *P*: FDR-corrected *P*-value for the Fisher's exact test in a 2 × 2 contingency table.

Regarding the large cluster containing abiotic responses, eight genes were shared by the response to heat and protein folding categories. A detailed exploration of these shared genes shows that five of them correspond to HSPs of the *HSP70 *(*At3g12580*, *At5g02490*, and *At3g09440*) and *HSP90 *(*At5g56010 *and *At5g56030*) gene families, both with activity as chaperones, one was a mitochondrial encoded chaperone (*At1g14980*), and two were luminal binding proteins (BiP) (*At5g42020 *and *At5g28540*) also characterized as chaperones [16]. Eight up-regulated genes were common to response to cold and to abscisic acid (ABA) stimulus; this was not surprising given the well established role of ABA in plant acclimation to low temperatures [17]. Furthermore, one of these shared genes, *At5g52310*, also appeared within the categories of responses to desiccation and hyperosmotic salinity. Another gene that promiscuously appears under different up-regulated GO categories was *At5g37770 *that encodes a protein with 40% similarity to calmodulin (CaM). This protein is involved in responses to mechanical stimulus, absence of light, cold, desiccation, hyperosmotic salinity, and ABA [18]. ABA has been described to affect the expression of CaM, illustrating the close relationship between hormones and phosphorilation and activiation of Ca^2+^-dependent kinases [18]. Two genes were in common between response to oxidative stress and toxin catabolism. Both genes encode for glutathione transferases. *At1g78380 *encodes GST8, (τGST gene family) whereas *At1g02930 *encodes GSTF6 (φGST gene family). GSTs are activated by several abiotic stresses and involved in herbicide detoxication [19]. Five up-regulated genes are shared between the ethylene stimulus response and response to metal ion categories. All these genes are members of the R2R3-MYB oncogene homologue family [20,21] also involved in the regulation of cell cycle, control of many aspects of plant secondary metabolism, and hypersensitive response cell death. Indeed, *At3g28910 *(*MYB30*) was also shared with the programmed cell death category.

Regarding the small cluster of up-regulated genes assigned to biotic stress categories, two genes, *At3g54230 *and *At1g74710*, were in the root of the cluster. The first gene encodes phytoalexin deficient 4 (*PAD4*), a lipase-like protein, involved in salicylic acid (SA) signaling and functions in gene-mediated and basal resistance. *PAD4 *interacts directly with other disease-resistance signaling proteins, like the enhanced disease susceptibility 1 protein (*EDS1*) [22]. Both proteins are recruited by Toll-interleukin-1 receptor (TIR)-type nucleotide binding-leucine rich repeat (NB-LRR) proteins to signal isolate-specific pathogen recognition [22,23]. The second gene encodes a protein with isochorismate synthase activity that is involved in SA biosynthesis [24].

Table 1 also contains two GO categories of under-represented genes. Five genes were related to organelle organization and biogenesis and three with DNA metabolism. A single gene was common to these categories, *At5g64630*, that encodes for the p60 subunit of the chromatin assembly factor 1 (*CAF1*) and is involved in the organization of shoot and root apical meristems and production of viable gametes [25].

### Functional categorization of genes under-expressed in TEV infected plants

**Figure 3:**
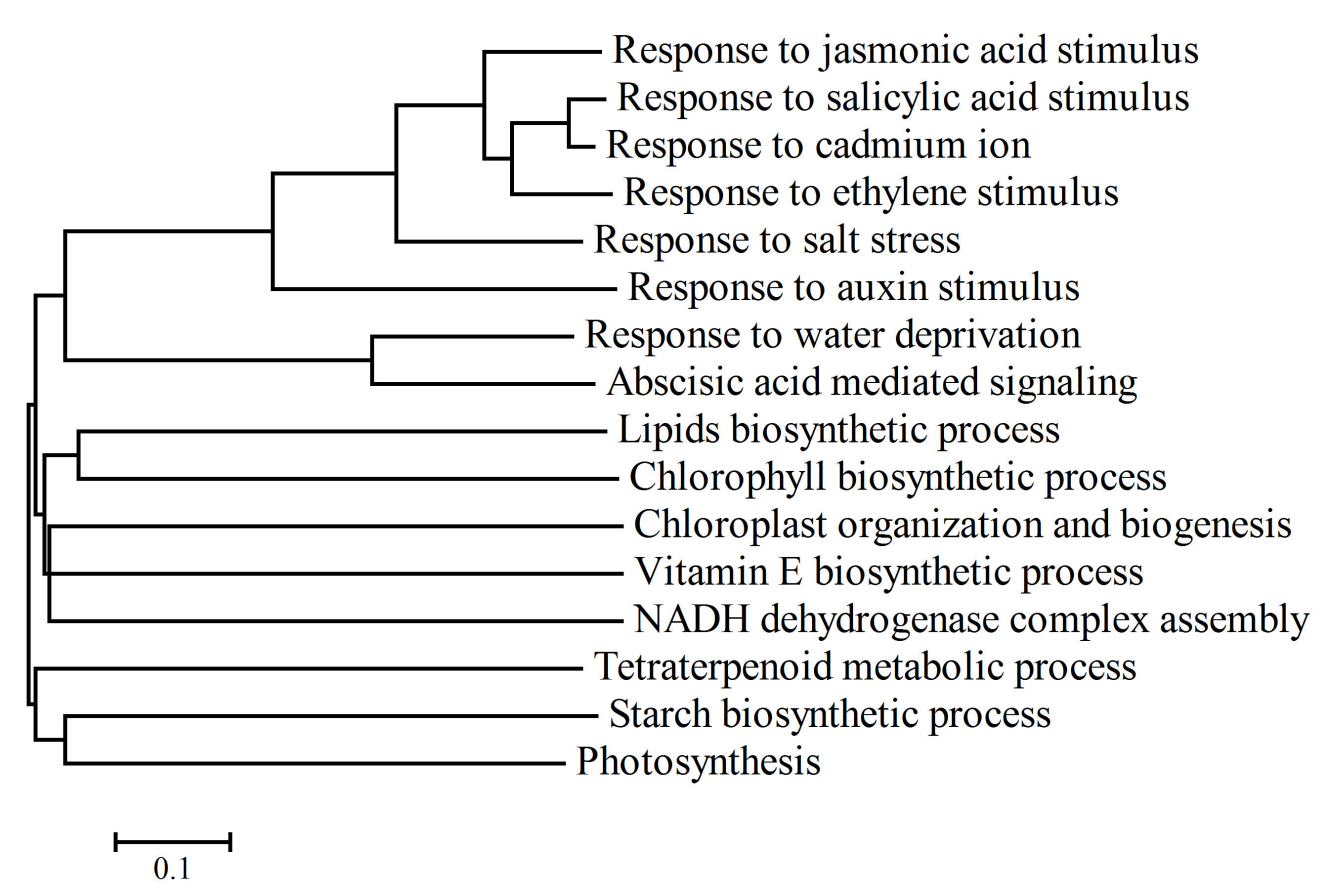
Neighbor-joining dendrogram illustrating the relationship among over-represented non-redundant GO categories obtained for genes that were down-regulated by TEV infection.

Sixteen non-redundant functional categories of down-regulated genes were over-represented in TEV infected plants; none was under-represented (Table 2). The most abundant category was constituted by genes involved in response to auxin stimulus (25) whereas the less abundant one contained the two genes involved in NADH-dehydrogenase complex (plastoquinone) assembly (*At1g74880 *and *At5g58260*). As in the previous case, we computed a neighbor-joining dendrogram relating these 16 non-redundant GO categories (Figure 3). Four categories did not share any gene with the other 12: chloroplast organization and biogenesis, vitamin E biosynthetic and tetraterpenoid metabolic processes, and NADH-dehydrogenase.

**Table 2:** Gene ontology analyses of down-regulated genes

**Non-redundant GO categories**	**Level**	**Differentially ** **expressed (%)**	**Total genes in** ** the class (%)**	***P***
**Over-represented**				
Photosynthesis	3	4.76 (16)	0.57	5.99×10-^7^
Response to jasmonic acid stimulus	4	3.79 (12)	0.98	8.94×10-^3^
Response to salicylic acid stimulus	4	3.15 (10)	0.89	3.70×10-^2^
Response to auxin stimulus	5	8.77 (25)	2.06	2.14×10-^6^
Response to salt stress	5	5.61 (16)	1.41	7.85×10-^4^
Response to water deprivation	5	4.21 (12)	0.87	1.59×10-^3^
Response to ethylene stimulus	5	4.21 (12)	1.23	1.97×10-^2^
Response to cadmium ion	6	3.95 (9)	0.44	3.67×10-^4^
Lipids biosynthetic processes	6	9.21 (21)	3.11	1.59×10-^3^
Chloroplast organization and biogenesis	6	2.63 (6)	0.36	1.84×10-^2^
Chlorophyll biosynthesis	7	4.52 (8)	0.20	1.03×10-^5^
Vitamin E biosynthesis	7	2.26 (4)	0.05	1.72×10-^3^
Abscisic acid mediated signaling	7	3.95 (7)	0.60	1.13×10-^2^
NADH dehydrogenase complex assembly	8	1.89 (2)	0.00	3.70×10-^2^
Starch biosynthetic processes	9	7.14 (4)	0.34	1.12×10-^2^
Tetraterpenoid metabolism	9	8.93 (5)	1.17	3.79×10-^2^

Non-redundant GO categories identified as enriched among up-regulated genes in infected plants versus mock-inoculated plants. The percentages of genes belonging to each category are reported for the differentially expressed genes and for the *A. thaliana *genes present in the microarray. The absolute number of genes is reported in parenthesis for the differentially expressed set. *P*: FDR-corrected *P*-value for the Fisher's exact test in a 2 × 2 contingency table.

Nine genes were shared among the responses to SA, Cd^2+^, ethylene, jasmonic acid (JA) and salt stress. Eight of them appear annotated in databases as MYB transcription factors (*At3g47600*, *At2g46830*, *At1g22640*, *At1g01060*, *At3g09600*, *At1g71030*, *At5g02840*, and *At5g59780*), the ninth one (*At5g59430*) corresponds to the telomeric repeat-binding protein 1 (*TRP1*), which also contains the typical MYB motifs [26]. Eight out of these nine genes were also included in the response to auxin stimulus, being *At1g71030 *(*MYBL2*) the missing one. *MYBL2 *has two peculiarities, first it only contains one of the typical two or three tryptophan repeats found in other MYB-like proteins and, second, it has a proline rich domain at the carboxi terminal end of the protein [27].

The functional categories of response to water deprivation and ABA-mediated signaling share six genes. Three of them were ABA-activated transcription factors (*At4g34000*, *At2g46680 *and *At1g45249*), *At4g33950 *is an ABA-activated protein kinase whose activity is triggered by osmotic stress, and the other two (*At3g11410 *and *At5g57050*) encode protein phosphatases 2C, which are negative regulators of ABA signaling [28].

Lipid and chlorophyll biosynthetic processes shared a down-regulated gene, *At4g15560*. This gene encodes the cloroplastos alterados 1 protein (*CLA1*) that has 1-deoxyxylulose 5-phosphate synthase activity required for the methylerythritol pathway, essential for chloroplast development in *Arabidopsis *[29].

Finally, photosynthesis and starch biosynthesis categories also shared a gene, *At3g55800*, that encodes for the chloroplastic sedoheptulose-1,7-biphosphatase (SBPase) involved in carbon reduction in the Calvin cycle [30]. Increases in SBPase expression have been associated to increases in photosynthetic activity and biomass production [30].

### Metabolic pathways altered upon TEV infection

Next, we sought to explore metabolic pathways that were associated with the level-5 GO functional categories, some of which have been described in the previous section. To do so, a matrix **Ω **was constructed quantifying the overlap between the 282 GO level-5 significant enriched functional categories and the 119 KEGG metabolic pathways. Each element in this matrix *ω*_*ij *_is the overlap score (defined in the Methods section) between the *i*th GO category and the *j*th metabolic pathway. A zero value means that not a single gene is present in both sets; the more overlap between both sets, the larger the index. The **Ω **matrix is reported in Additional file 1. As a first preliminary analysis, we studied which GO categories included more KEGG pathways. Columns in the matrix (i.e., KEGG pathways) were added up to compute a column vector of scores. The elements of the vector were then rank-ordered. The top 2.5% elements in this vector corresponded to the seven GO categories that overlapped the most with KEGG pathways. Not surprisingly, these GO categories contained KEGG metabolic pathways related with basal carbon metabolism. These functional categories were, sulfur compound biosynthesis, carbohydrate metabolism, carboxylic acid metabolism, carbohydrate catabolism, alcohol catabolism, response to oxidative stress, and energy derivation by oxidation of organic compounds (Additional file 1). The first three categories were over-represented whereas the remaining four were under-represented.

Focusing in GO functional categories related to biotic stress, the innate immune response, which was an over-represented category, was ranked 213/282 and it was related to 16 KEGG metabolic pathways (Additional file 1). Most of these pathways correspond to amino acid synthesis (K, W, G. S, V, and L) or secondary metabolism (e.g., methane metabolism, phenylpropanoid biosynthesis, ether lipid metabolism, benzoate degradation, and fatty acid metabolism). The response to bacterium, an under-represented category, was ranked 226/282 and overlapped with 14 KEGG pathways, including again amino acid metabolism, secondary metabolism (methane metabolism, phenylpropanoid biosynthesis, ether lipid metabolism, benzoate degradation), nitrogen metabolism, oxidative phosphorilation and nicotinate and nicotinamide metabolism.

Focusing now on non-biotic stresses, the response to heat over-represented category was ranked 170/282 and overlapped with five KEGG pathways: glycolysis and gluconeogenesis, fructose and mannose metabolism, carotenoid biosynthesis, ascorbate and aldarate metabolism, and arginine and proline metabolism. Response to ABA ranked 214/282 and overlapped with 16 KEGG pathways. These pathways were diverse and ranged from central metabolism (glycolysis), secondary metabolism (pyruvate and sulfur metabolism), to detoxification pathways (e.g., γ-hexachlorocyclohexane, naphthalene and anthracene degradations). The response to ethylene stimulus ranked 144/282 and overlapped with only five KEGG pathways: aminosugars metabolism, methane metabolism, phenylpropanoid metabolism and amino acid (F, Y, and W) metabolism.

## Discussion

Viruses alter the transcriptional networks of their host cells. Some of these alterations may directly have an impact in the virus' replication, cell to cell and systemic spreads, and accumulation while others may simply be side-effects of virus replication. Similarly, many of these alterations may be related with disease development. Therefore, identifying which genes change their expression as a consequence of virus infection provides invaluable information to identify the host processes involved in virus replication. Here we have used DNA microarrays to investigate the effect of TEV, a model system among the postyviruses, on the transcriptome of the susceptible host *A. thaliana *ecotype L*er *[12,13]. This approach has allowed us to simultaneously analyze the response of 28,964 protein-coding gene transcripts and 87 miRNAs to TEV infection. The 1027 genes identified as up-regulated by TEV infection and the 700 genes identified as down-regulated by TEV infection provide candidate genes for further investigation of the interaction of this important virus and their hosts.

### Alterations in primary metabolism and cell cycle

Genes involved with chloroplast biogenesis and activity were under-represented among the over-expressed genes. This list includes genes involved in chlorophyll biosynthesis and carbon fixation, suggesting a possible reason for the appearance of chlorosis and stunting in the TEV-infected *A. thaliana *plants.

Genes involved in DNA metabolism were also under-represented among genes over-expressed in infected plants. *At3g19150*, corresponds to the Kip-related protein gene (*KRP*) that encodes a cyclin-dependent kinase inhibitor which acts as negative regulator of cell division. Thus, under-expressing this gene may speed up cell division. The second gene, *At5g04560*, encodes the DME DNA glycosylase that activates the maternal *MEA *allele in the endosperm. The third gene, *At5g64630*, encodes for the p60 subunit of the *CAF1 *factor that is required for cell differentiation. Thus, all in all, under-expression of these genes may affect cell division and differentiation.

### Alteration in antiviral responses

Many genes over-expressed by TEV infection were stress- and defense-related genes. One of these over-expressed genes was *PAD4*, which is involved in signaling during plant defense responses. This gene was also shown to be over-expressed after infection of *A. thaliana *with several other viruses, including cucumoviruses, potexviruses, potyviruses, and tobamoviruses [3], suggesting that it may be a general response to virus infection rather than a TEV specific response. *PAD4 *(along with many other genes, e.g., *BG2*, *PR1*, *PR5*, and *PAD3*) is controlled through signaling pathways that involve SA. We found the SA pathway being one of the most altered GO category after TEV infection, with certain genes being over-represented among the altered GO categories and some under-represented.

R2R3-MYB constitutes the largest MYB gene family in plants [21]. These transcription factors participate in many different cellular processes, from the regulation of secondary metabolism, to control of development and to determination of cell fate and identity. Interestingly, accumulated evidences suggest that they are often involved in combinatorial interactions with other transcription factors for the generation of highly specific expression patterns [21]. They are also involved in plant response to environmental stresses and their expression is strongly correlated with cell death during the hypersensitive response to pathogen attack, including the hypersensitive response for which they act as positive regulators [31]. Upon TEV infection, the response of MYB genes was quite variable, and ranged from under-expression of *TRP1 *and *MYBL2 *genes (involved in SA- and JA-mediated responses to pathogens) to over-expression of genes involved in ethylene stimulus response and response to metal ion categories.

One of the more interesting responses to TEV infection was the over-expression of genes related to protein-folding and thermal stress. HSPs are well known to be over-expressed after viral infection either as part of a more general stress response or actively induced by the virus in its own benefit. Supporting the first possibility, Jockush *et al*. [32] reported that tobacco plants expressing mutant TMV coat proteins triggered the over-expression of HSP as a consequence of the presence of large amounts of denaturized proteins in the cytoplasm. Alternatively, viruses may elicit HSP expression via specific mechanisms. The over-expression of HSP has been frequently observed in response to both plant and animal viruses [33,34] suggesting that these proteins may be required for virus replication or used as an extrinsic buffering mechanism to cope with the high mutational load produced during virus low-fidelity RNA virus replication [35].

For example, *HSP101 *enhances the translation of mRNAs in yeast and has been speculated that could also be a factor involved in tobamovirus replication [3]. In summary, our results add extra support to the view that HSP over-expression is an unspecific response to viral infection and not a particular feature of TEV infection.

Adaptive responses to abiotic stresses were classically associated to ABA signaling; while SA-, JA- and ethylene-mediated responses played major roles in disease resistance. However, experimental data have shown that reduced ABA levels correlated with increased resistance to different pathogens likely by its interaction with ethylene and JA pathways [36]. Consistently with this observation, several ABA-activated transcription factors and an ABA-activated protein kinase have been down-regulated in plants infected with TEV.

It has been well established that symptoms in potyvirus-infected plants are associated with the RNA silencing suppressor activity of the HC-Pro protein that interferes with the endogenous miRNA functions, causes misregulation of the expression of several miRNA-regulated transcription factors and produce developmental defects [37]. However, none of the 87 miRNAs spotted in the chip showed significant alteration in concentration in infected plants, thus suggesting that this approach would not be suitable for identifying miRNA-regulated genes.

## Conclusion

The data presented in this study demonstrates the varied effects at the transcriptomic level of TEV infection on a susceptible host. The observed changes in gene expression of genes involved in biotic and abiotic stress responses may be either directly or indirectly responsible for the mild symptoms developed by infected plants. None of the observed alterations in *A. thaliana *gene expression can be specifically associated to TEV infection but, instead, represent general responses to stress-induced by virus infection. Nonetheless, this type of experiments specifically designed to characterize host responses to viral infection might contribute to elucidating the mechanisms underlying plant defense responses to virus infection.

## Methods

### Virus and plants

The infectious clone pTEV-7DA (GeneBank DQ986288) was kindly provided by Prof. J.C. Carrington (Oregon State University). This clone contains a full-length cDNA of TEV and a 44 nt long poly-T tail followed by a *Bgl*II restriction site cloned into the pGEM-4 vector downstream of the SP6 promoter. 5' capped infectious RNA was obtained upon transcription of *Bgl*II-digested pTEV-7DA using SP6 mMESSAGE mMACHINE kit (Ambion). All other basic procedures are described elsewhere [38]. Three weeks-old *A. thaliana *L*er *plants were inoculated with 5 μg RNA. Afterwards, plants were maintained in the greenhouse at 25°C and 16 h light. Successful infections were confirmed by Western blot hybridization analysis 21 dpi using commercial antibodies anti-coat protein conjugated with horse-radish peroxidase (Agdia).

### RNA extraction and microarray hybridization

Total RNA was extracted from control (mock inoculated) and systemic infected plants, and used in an amplification reaction with the MessageAmp II aRNA Amplification kit (Ambion) following manufacturer's instructions.

Five replicates for each sample category were generated. RNAs from each individual sample were extracted and amplified. A global reference was generated by equimolarly mixing amplified RNAs from each of the 10 samples. Amplified RNA from each individual sample, plus the reference, were used for labeling. For each category, three samples were labeled with Cy5 and two with Cy3, and compared with the corresponding reversed-labeled reference sample. Long 70-mers oligonucleotide microarrays contain 29,110 probes from the Operon Arabidopsis Genome Oligo Set Version 3.0 (Operon). This oligo set represents 26,173 protein-coding genes, 28,964 protein-coding gene transcripts and 87 miRNAs and is based on the ATH1 release 5.0 of the TIGR *Arabidopsis *genome annotation database  and release 4.0 of the miRNA Registry at the Sanger Institute . Oligos were rehydrated and DNA was immobilized by UV irradiation. Slides were then washed twice in 0.1% SDS, 4 times in water, and then dipped in 96% ethanol for 1 min and dried by centrifugation. Slides were prehybridized 30 min at 42°C with 100 μL of 6 × SSC, 1% BSA and 0.5% SDS, under a 60× 22 mm coverslip LifterSlip (Erie Scientific) in an ArrayIt microarray hybridization cassette (TeleChem). Slides were then rinsed five times in H_2_O and dried by centrifugation. Slides were hybridized immediately. Labeled RNA was used to hybridize the slides basically as described in [39]. After hybridization and wash, slides were scanned at 532 nm for the Cy3 and 635 nm for the Cy5 dyes, with a GenePix 4000B scanner (Axon Molecular Devices), at 10 nm resolution and 100% laser power. Photomultiplier tube voltages were adjusted to equal the overall signal intensity for each channel, to increase signal-to-noise ratio, and to reduce number of spots with saturated pixels. Spot intensities were quantified using GenePix Pro 6.0 (Axon Molecular Devices).

### Microarray data analysis

Spots with a net intensity in both channels lower than the median signal plus twice standard deviations were removed as low signal spots. Data were normalized by median global intensity and with LOWESS correction [40] using the Acuity 4.0 software (Axon Molecular Devices). Finally, only probes for which a valid data was obtained in at least seven out of the ten slides were considered for further analysis (13,722 spots). Median, mean and SEM values were calculated from each treatment (control and TEV-infected plants), and all data were normalized to the median of the expression in control samples. To detect differentially expressed genes in plants infected with TEV compared to uninfected plants, data were analyzed with the SAM package [14], using a 5% FDR with no fold-change cut-off. Gene lists were further analyzed with FatiGO to find differential distributions of gene ontology (GO) terms between statistically differential genes and the rest of genes in the microarray (Fisher's exact test in 2 × 2 contingency tables), with *P *values adjusted after correcting for multiple testing [15]. Gene descriptions were downloaded from TAIR database .

The starting point for identifying under- and over-expressed metabolic pathways from gene expression data are the 119 *A. thaliana *pathways available in the January 2008 release of KEGG database [41]. These pathways contained, in average, eight enzyme-coding genes per pathway. The 284 groups of functionally equivalent genes (at level 5) identified by FatiGO contained each an average of 50 genes. Subsequently, every pathway and group were scored by computing the log_2 _of the ratio between the gene expression level in TEV-infected plants and control plants (mock inoculated) and normalized by the number of genes in the set. To minimize the number of false positives, only the expression ratios under 0.7- or over 1.3-fold change were allowed to contribute to the scoring function. The pathways and GO groups with lower or higher scores were selected. To determine the statistical significance of these scores, sets of genes were randomly selected and their scores computed. For GO groups, the sets contained 50 genes, and for the KEGG pathways, the set contained an average of eight genes. Next, we defined the degree of overlapping between KEGG pathways and GO functional categories as the ratio between the intersection and the union of genes from presents in both sets. Finally, the statistical significance of this overlap statistic was assessed by bootstrapping the vector of values obtained for each GO functional category.

Microarrays were deposited at NCBI Gene Expression Omnibus database under accession number GSE11088.

## Competing interests

The authors declare that they have no competing interests.

## Authors' contributions

PAR and PdlI did all the plant work. PAR and PC did the RNA extractions, labeling and microarray hybridizations. MAPA analyzed the microarray data and supervised microarray work. JC, GR and AJ developed the algorithm and analyzed the overlap between GO categories and KEGG pathways. SFE conceived and designed the experiments, analyzed the data and wrote the manuscript. All authors discussed the results and commented on the manuscript.

## Supplementary Material

Additional file 1 Table s1. Supplemental table 1. Click here for file
